# A comprehensive analysis of the effects of *DGAT1* K232A polymorphism on milk production and fertility traits in Holstein Friesian and Jersey cows reared in Türkiye

**DOI:** 10.5194/aab-67-455-2024

**Published:** 2024-10-02

**Authors:** Sena Ardicli, Ozden Cobanoglu, Ertugrul Kul, Samet Hasan Abaci, Eser Kemal Gurcan, Soner Cankaya

**Affiliations:** 1 Department of Genetics, Faculty of Veterinary Medicine, Bursa Uludag University, 16059 Bursa, Türkiye; 2 Department of Animal Science, Faculty of Agriculture, University of Kirsehir Ahi Evran, 40200 Kırşehir, Türkiye; 3 Department of Animal Science, Faculty of Agriculture, University of Ondokuz Mayis, 55139 Samsun, Türkiye; 4 Department of Animal Science, Faculty of Agriculture, University of Namik Kemal, 59030 Tekirdağ, Türkiye; 5 Department of Sport Management, Faculty of Yasar Doğu Sport Sciences, University of Ondokuz Mayis, 55139 Samsun, Türkiye

## Abstract

Research on the diacylglycerol acyltransferase 1 (*DGAT1*) K232A marker in cattle shows inconsistent results across regions, largely due to small sample sizes, limited genetic variation, and data restricted to few lactations, which complicates establishing a reliable genotype–phenotype correlation. This research aimed to determine the effect of the K232A polymorphism of the bovine *DGAT1* gene on milk production and quality traits in dairy cattle. We used 1104 cattle, including 828 Holstein Friesian and 276 Jersey cows. The analysis utilized extensive data from six lactations of cows raised on four commercial dairy farms. We genotyped the population using the polymerase chain reaction–restriction fragment length polymorphism (PCR-RFLP) technique and Sanger sequencing for verification. We then evaluated the 305 d and test-day milk yields as well as fat and protein yields and percentages. The number of inseminations per conception and calving ease were also assessed as reproduction indices. Genotype–phenotype associations were quantified using linear mixed models. The AA genotype was absent in Jersey cows, and the heterozygous genotype was predominant in both breeds. The K232A marker was significantly associated with test-day milk yield, fat, and protein content in Jersey cows. Further, it substantially affected the fat percentage of milk in Holstein Friesian cows ( p<0.001 ). We found that the KK genotype is highly desirable for milk quality and especially fat content. This comprehensive assessment demonstrated that the KK genotype of the *DGAT1* K232A polymorphism significantly influenced fat and protein contents in dairy cattle.

## 1 Introduction

Milk production is a phenotypic trait determined by the genotype and the environment that shows a large degree of variability in dairy cows. Many genes with additive effects determine the quantity and quality of milk; thus, these traits follow a polygenic inheritance model. By choosing the genes thought to be related to milk production and the appropriate genotypes of these genes, it will be possible to make more accurate predictions of genetic values for selection candidates and speed up the selection process.

**Figure 1:**
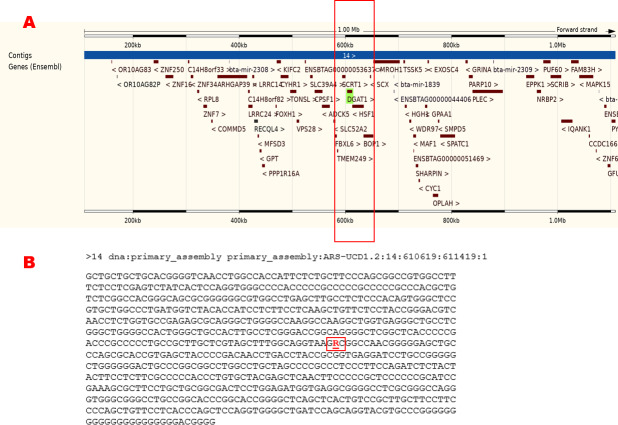
Brief information about the region in detail for the bovine diacylglycerol O-acyltransferase 1 (*DGAT1*), primary_assembly 14:603813–612791 (forward strand; ARS-UCD1.2:CM008181.2). **(a)** The genomic location, that is, the nearest genes of the *DGAT1* gene in the cattle genome. **(b)** The flanking sequence represents the *DGAT1* K232A variant (rs109234250). The figure was created using the Ensembl genome browser (2024; https://www.ensembl.org/Bos_taurus/Gene/Summary?db=core;g=ENSBTAG00000026356;r=14:603035-612781, last access: 27 September 2024).

Several researchers have focused on the BTA14 regarding the connection between particular quantitative trait loci (QTLs) and milk yield/composition traits – primarily fat content (Ashwell et al., 2004; Fink et al., 2020; Weller et al., 2003). Thus, many candidate genes associated with bovine milk production traits have been identified in recent decades (Fig. 1a). One of the most famous genes located on BTA14 is the diacylglycerol acyltransferase 1 (*DGAT1*) gene (GenBank accession number AJ318490). This gene significantly affects metabolism and especially intestinal fat absorption, adipose tissue formation, and lipoprotein assembly (Mohammed et al., 2015). The bovine *DGAT1* encodes an enzyme with the same name (DGAT1), which catalyzes the final step in triglyceride synthesis (Kong et al., 2007; Thaller et al., 2003). This multipass transmembrane protein acts as a crucial metabolic enzyme, converting diacylglycerol and fatty acyl coenzyme A to triacylglycerol (Lehner and Kuksis, 1996). The gene *DGAT1*, responsible for encoding this biochemically important enzyme, is expressed in almost all tissues, including mammary glands (Mahmoudi and Rashidi, 2023). It is on the centromeric region of BTA14 (Grisart et al., 2002; Mahmoudi and Rashidi, 2023; Winter et al., 2002), located in the 603813-612791 forward strand, and consists of 17 exons and 16 introns (Ensembl, 2024). Several studies reported a strong association between the bovine *DGAT1* and various milk-related traits in dairy cattle (Anton et al., 2012; Ardicli et al., 2018; Bobbo et al., 2018; Gothwal et al., 2022; Grisart et al., 2002, 2004; Winter et al., 2002).

Concerning the bovine *DGAT1* gene sequence, the dinucleotide change (AA/GC) at positions 10433 (g.10433G > A) and 10434 (g.10434C > A) in exon 8 leads to a non-conservative substitution of lysine by alanine at amino acid position 232 (Gothwal et al., 2022). Increased milk content in different breeds is strongly associated with the lysine position 232 of the protein encoded by bovine *DGAT1*; an alanine at this position is associated with lower milk fat content (Winter et al., 2002). The origin of this causal mutation (Mahmoudi and Rashidi, 2023) can be traced back to cattle domestication (Grisart et al., 2002; Winter et al., 2002). The lysine encoding variant is the ancestral constituent of *DGAT1*. The K232A alteration existed in the early stages of, or even before, bovine domestication (Winter et al., 2002). The presence of an alanine variant in the indigenous Anatolian Black cattle breed points out clues for the history of this alteration because these cattle have been living at the site of the domestication of European *Bos taurus* (Medjugorac et al., 1994). The subspecies *Bos taurus indicus*, which was domesticated separately, does not carry the alanine variant (Loftus et al., 1994). This mutation plays a critical role in triacylglycerol metabolism by affecting the activity of the resultant enzyme (Grisart et al., 2002; Mahmoudi and Rashidi, 2023; Winter et al., 2002). Many researchers have demonstrated that *DGAT1* K232A polymorphism has impacts on milk yield (Anton et al., 2008; Ardicli et al., 2018; Bovenhuis et al., 2016; Gothwal et al., 2022; Li et al., 2021; Manga et al., 2011; Mao et al., 2012; Samuel et al., 2023; Schennink et al., 2007; Vanbergue et al., 2016), fat content (Carvajal et al., 2016; Li et al., 2021; Mahmoudi and Rashidi, 2023; Manga et al., 2011; Mao et al., 2012), and protein content (Anton et al., 2008; Li et al., 2021; Mahmoudi and Rashidi, 2023; Mao et al., 2012; Vanbergue et al., 2016). On the other hand, several studies reported contrary results on milk-related traits (Akyuz et al., 2015; Ardicli et al., 2018; Khan et al., 2019; Krovvidi et al., 2021; Manga et al., 2011; Strzałkowska et al., 2005; Tumino et al., 2021; Valenti et al., 2019; Van Gastelen et al., 2017).

Although the *DGAT1* K232A marker has been widely studied in various cattle breeds, the results from different geographical regions often conflict even for the same traits. In many studies, it is striking that some limiting factors prevent the establishment of a reliable genotype–phenotype balance. First, several studies have been conducted on limited sample sizes. Moreover, genotyping dairy populations with insufficient sires results in low genetic variation. The absence of particular genotypes causes ignoring these variants' critical effects. Predominantly, a vast majority of the studies consist of data limited to a few lactations. On the other hand, some essential environmental impacts, such as lactation curve constituents or calving season, are lacking in the statistical analysis. Therefore, we aimed to quantify the effects of the *DGAT1* K232A polymorphism (rs109234250) on milk production traits in Holstein Friesian and Jersey dairy cows.

## 2 Material and methods

### 2.1 Animal material and phenotypic records

Animals used in the present study ( n=1104 ) were purebred Jersey ( n=276 ) and Holstein Friesian cows ( n=828 ). All of the Jersey cows were from one and Holstein Friesians were from three commercial dairy herds in the south Marmara region of Türkiye. All animals were raised in free-stall barns and milked two (for Jerseys) and three times (for Holstein Friesians) a day. Cows were milked in parlors fitted with electronic systems that automatically recorded the milk volume. Thus, the milk yield for each cow during each milking session was tracked using DeLaval's Alpro herd management system (DeLaval International, Tumba, Sweden). The mean results of each cow from two (for Jerseys) and three (for Holstein Friesians) consecutive milkings were used. Milk samples were taken individually on milk test days monthly for six lactations. Then, 305 d milk yield (305-DMY) was calculated using the individual daily milk yield record dataset. Thus, we acquired the test-day milk yield (TDMY) and 305-DMY records. From the monthly milk control samples, we obtained data on milk composition traits, including fat content (FC), protein content (PC), test-day fat yield (TDFY), test-day protein yield (TDPY), 305 d fat yield (305-DFY), and 305 d protein yield (305-DPY). Milk composition analysis was evaluated by infrared analysis with a Bentley 2000 mid-infrared instrument (Bentley Instruments, Chaska, MN, USA). The data were obtained from three different herds for the Holstein Friesian breed, whereas the data on Jerseys originate from a single farm. We obtained the number of inseminations per conception (NI) and calving ease records (CE) from herd 3 ( n=361 ). CE was evaluated on a scale according to the following criteria: (1) unassisted calving, (2) calving requiring remote assistance, (3) calving with help, and (4) caesareans applied. Since there were low numbers of animals with category 4, we combined categories 3 and 4 and coded them both as 3 to minimize the severe category problem, as González-Recio et al. (2007) suggested. Regarding the entire population, we gathered lactation rank, calving season, calving year, and days in lactation records for the subsequent quantitative analysis. We excluded the records with missing sire identification and incorrect calving dates from the study. This study complied with the relevant national regulations and institutional policies for the care and use of animals (approval number 2010/6-05).

### 2.2 DNA extraction and genotyping

We isolated the genomic DNA from whole blood samples ( ∼ 10 mL) taken into K ${}_{3}$ EDTA tubes (Vacutest Kima SRL, Italy) from the vena jugularis of the animals. All blood samples were conserved at - 20 °C until DNA extraction. We then obtained the purified DNA according to the standard phenol–chloroform–isoamyl alcohol ( 25:24:1 ) method as Green (2012) suggested. DNA concentration (ng mL ${}^{-1}$ ) and absorbance ratio ( 260/280 value) were measured using a NanoDrop spectrophotometer (NanoDrop 2000c, Thermo Fisher Scientific, Wilmington, DE, USA).

The genotyping of *DGAT1* K232A polymorphism in exon 8 (Fig. 1b) was performed by the polymerase chain reaction–restriction fragment length polymorphism (PCR-RFLP) method and Sanger sequencing. A touch-down PCR protocol was applied with the bovine *DGAT1* primer sequences to amplify a 411 bp amplicon, as shown in Table 1. PCR mixture consisted of 50 µ M primer (1 µ L from each forward and reverse primer), 5 µ L (25mM) MgSO ${}_{4}$ , 1 µ L dNTP (200 µ M), 5 µ L buffer (10x), 1 µ L Taq DNA polymerase enzyme, and 33.5 µ L ddH ${}_{2}$ O. We used ∼ 2.50 mL (70–95 ng) genomic DNA for the amplification. Consumables were obtained from Biomatik (Biomatik, Cambridge, Canada) and Thermo (Thermo Fisher Scientific, Inc., Waltham, MA, USA). After PCR, products were electrophoresed using 2 % agarose gels (Sigma Aldrich, Steinheim, Germany) in Tris–Borate–EDTA (TBE) (1 × ).

**Table 1:** Primers sequences (from 5 ${}^{\prime }$ to 3 ${}^{\prime }$ ) and restriction enzyme used for genotyping the *DGAT1* locus in the current study.

Primer sequences ${}^{1}$	Amplicon size (bp)	PCR conditions ${}^{2}$	Restriction enzyme	Digestion product size (bp)
F: GCACCATCCTCTTCCTCAAG	411	94 °C 4 ${}^{\prime }$ , 10 cycles (94 °C 60 s, 66 °C 60 s; - 1 °C per cycle, 72 °C 60 s),	*Eae*I	KK: 411
R: GGAAGCGCTTTCGGATG		72 °C 15 ${}^{\prime }$ , 25 cycles (94 °C 60 s, 56 °C 120 s, 72 °C 60 s)		KA: 411, 208, 203
				AA: 208, 203

${}^{1}$ Primer sequences were used as suggested by Winter et al. (2002). ${}^{2}$ A touch-down PCR protocol was used to amplify the 411 bp PCR product. *DGAT1*: diacylglycerol acyltransferase 1.

In total, 15 µ L of PCR product were digested with 0.50–1 mL of the *Eae*I restriction enzyme (New England Biolabs (NEB), Ipswich, MA, USA, or Thermo Fisher Scientific). We prepared the enzyme–buffer mix consisting of 1 µ L restriction enzyme with 8 µ L of the respective buffer and 10 µ L of the PCR product. The incubation was modified from 3 h to overnight. Amplifications and enzyme incubations were run on two thermal cyclers (Palm Cycler GC1-96, Corbett Research, Sydney, Australia, and T100 Thermal Cycler, Bio-Rad, USA). The digested fragments were separated through 3 % agarose gel electrophoresis and visualized under an automatic gel documentation system (DNR MiniLumi Bio-Imaging Systems, Israel). The predicted bands for each genotype are listed in Table 1.

For further confirmation, we sequenced representative samples from each genotype ( n=21 ) for the bovine *DGAT1* gene (GenBank accession number AY065621, genomic sequence NC_037341.1, chromosome 14, reference ARS-UCD2.0 primary assembly). To amplify the target, PCR was performed as mentioned above. The amplified PCR amplicons were purified using the DNA Clean and Concentrator kit (Zymo Research, Orange, California). We sequenced the products following the manufacturer's instructions using the SeqStudio™ Genetic Analyzer with SeqStudio™ Data Collection Software automatic sequencer (Applied Biosystems, Carlsbad, CA). Ultimately, the results were compared and confirmed by the cattle genome provided by the Ensembl genome browser (https://www.ensembl.org/Bos_taurus/Gene/Summary?db=core;g=ENSBTAG00000026356;r=14:603813-612791;t=ENSBTAT00000037423, last access: 27 September 2024).

### 2.3 Statistical analysis

Allele and genotype frequencies of the *DGAT1* K232A polymorphism were calculated based on the suggestions by Falconer and Mackay (1996). The appropriateness to the Hardy–Weinberg equilibrium (HWE) was determined with chi-square ( $\chi^{2}$ ) of observed and predicted genotypic frequencies. If the genotype count was below five, we used Fisher's exact test to evaluate the HWE. Population genetic parameters, including heterozygosity (He), effective allele numbers (Ne), and polymorphism information content (PIC), were calculated according to the formulas stated by Nei and Roychoudhury (1974) and Botstein et al. (1980). We used linear mixed models, selected based on the coefficient of determination ( $R^{2}$ ) values, in the analysis of variance (ANOVA) to assess the genotype–phenotype association. Minitab (PA, USA, v17.1.0) statistical software was used for the association analysis. The statistical model was as follows:

$$Y_{ijklmnop}=\mu +A_{i}+B_{j}+C_{k}+D_{l}+\beta \;E_{m}+F_{n}+\;G_{o}+e_{ijklmnop}$$

where $Y_{ijklmnop}$ is the phenotypic record, μ is the overall mean, $A_{i}$ is the fixed effect of the i th calving year ( i=2010 , 2011, 2012, 2013, 2014, 2015), $B_{j}$ is the fixed effect of the j th calving season ( j is autumn, winter, spring, and summer), $C_{k}$ is the fixed effect of k th lactation rank ( k=1 , 2, 3, 4, 5, and 6), $D_{l}$ is the fixed effect of the l th *DGAT1* genotype ( l is KK, KA, and AA), β $E_{m}$ is the regression effect of days in lactation, $F_{n}$ is the impact of animal factor ( n is the varying performance value of an individual cow in different lactations), $G_{o}$ is the fixed effect of the o th herd ( o=1 , 2, and 3), and $e_{ijklmnop}$ is the random error.

Initially, the sire effect was included in the model. However, it was excluded because of the minimal number of genotyped bulls that were progenies of the same sire, as Curi et al. (2006) indicated. The model included the herd effect because of three different herds for the Holstein Friesian breed (the data on jerseys originate from a single farm). Furthermore, the model consists of only two genotypes to evaluate the NI and CE since the AA genotype was absent in herd 3. Genotype–environment interactions were either insignificant or, in some cases, meaningless for interpreting the genotype-driven interaction due to the overwhelming environmental influence (e.g., the genotype × lactation rank). Thus, they were not considered further. We analyzed genotypes and milk production traits using least-squares means (LSMs) and their corresponding standard errors (SEs). When significant associations were observed, differences between groups were analyzed using Tukey's post hoc test.

We estimated additive and dominance effects based on a reparameterized model as demonstrated by Falconer and Mackay (1996). In this respect, the additive effect is the difference between the means of two homozygous divided by 2, while the dominance effect is the deviation of the heterozygote from the mean of the two homozygotes.

## 3 Results

### 3.1 The identification of the single-nucleotide polymorphism (SNP)through genotyping 

**Figure 2:**
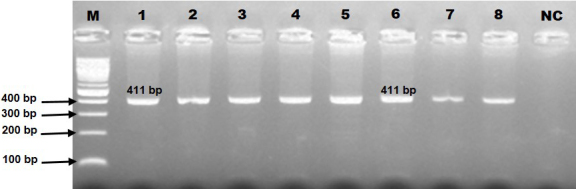
The electrophoresis pattern of K232A polymorphism (rs109234250) within the bovine *DGAT1* concerning PCR amplification (amplicon size of 411 bp). NC: negative control.

**Figure 3:**
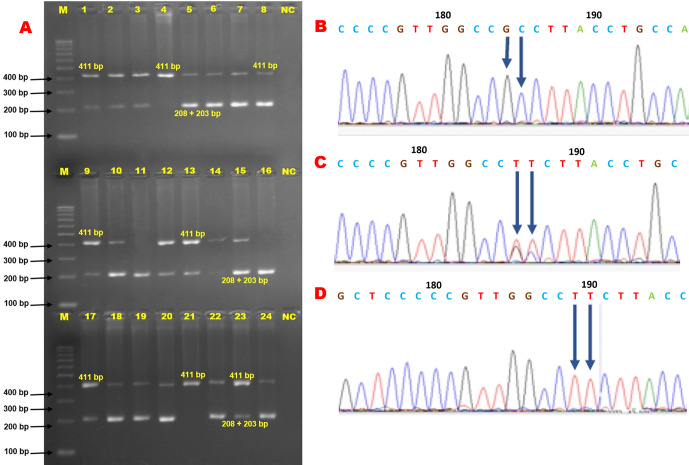
Results of PCR-RFLP and Sanger sequencing for bovine *DGAT1* K232A polymorphism. **(a)** The electrophoresis pattern of restriction enzyme digestion of PCR product with *Eae*I for K232A polymorphism within the bovine *DGAT1* gene. Lines 4 and 21: KK; lines 1–3, 5–10, 12–15, 17–20, and 22–24: KA; lines 11 and 16: AA; NC: negative control. Considering the AA genotype, the diagnostic bands (208 and 203 bp) appear to be a single band (208 + 203 bp) because of their closeness. **(b)** Chromatograph of the K232A locus of the *DGAT1* gene representing the AA homozygous genotype. **(c)** Chromatograph of the K232A locus of the *DGAT1* gene representing the heterozygous genotype. **(d)** Chromatograph of the K232A locus of the *DGAT1* gene representing the KK homozygous genotype. The dinucleotide change (AA/GC) at positions 10433 and 10434 in exon 8 leads to a non-conservative substitution of lysine by alanine at position 232. The two alleles at this locus are AA and GC, encoding lysine (K) and alanine (A) at the amino acid position 232. These two alterations in the *DGAT1* gene lie immediately adjacent to each another.

In genotyping the K232A substitution in the bovine *DGAT1* gene (primary_assembly 14:611019 (forward strand)) using *Eae*I restriction enzyme, the approach targets the combined dinucleotide change at positions 10433 and 10434 from AA to GC, which corresponds to the amino acid substitution from lysine to alanine at position 232. The restriction enzyme recognizes a specific sequence, cutting only if this sequence matches the GC variant, thereby indicating the presence of alanine, while the AA sequence, indicative of lysine, remains uncut. In this context, the 411 bp PCR product (Fig. 2) harboring K232A polymorphism of the bovine *DGAT1* gene was discriminated by the *Eae*I restriction enzyme (Y/GGCCR) digestion. The distinct banding characterized the KK genotype by the single undigested band (411 bp). The three-band pattern (411, 208, and 203 bp) was diagnostic for the heterozygote genotype. AA is distinguishable by two bands (208 and 203 bp). Notably, these two bands appear to be a single band (208 + 203 bp) because they are very close (Fig. 3a). However, this does not pose any difficulties in diagnosis. The three genotypes (KK, KA, and AA) were observed in Holstein Friesian cows, but AA was absent in Jerseys (Fig. 3). Following PCR-RFLP, we sequenced the PCR amplicons with a genetic analyzer. The chromatograph representing respective genotypes is shown in Fig. 3b–d. Consequently, the dinucleotide transition from AA to GC at positions 10433 and 10434 (AJ318490.1) in exon 8, which results in a non-conservative amino acid substitution, replacing lysine with alanine at position 232, was properly analyzed.

### 3.2 Genetic diversity parameter estimates

Table 2 shows the genotypic distribution, population genetic parameters, and HWE test results. The results are presented separately for both breeds and the whole population. Among 1104 cows, there were only seven animals with the AA genotype. This genotype was absent in the Jersey breed. The frequency of heterozygous animals was predominant in both breeds.

On the other hand, Jersey cows exhibited the highest frequency of the K allele (0.59). We observed concordance with HWE in neither a specific breed nor the entire population. He and Ne values were higher in Holsteins. We also observed the highest PIC in this breed, as shown in Table 2. For both breeds, the marker showed mild variability and informativeness.

**Table 2:** Genotype and allele frequencies, Hardy–Weinberg equilibrium (HWE) test results, and population genetics parameters of the bovine *DGAT1* K232A polymorphism in the Holstein Friesian ( n=828 ) and Jersey ( n=276 ) breeds and in the entire dairy population ( n=1104 ).

Breed	Number of cows			Genotypic			Allelic		HWE test ${}^{1}$	He	Ne	PIC
	per genotype			frequency (%)			frequency					
	KK	KA	AA	KK	KA	AA	K	A				
Holstein Friesian	56	765	7	6.76	92.39	0.85	0.53	0.47	p<0.001	0.4982	1.9928	0.3741
Jersey	49	227	0	17.75	82.25	0	0.59	0.41	p<0.01	0.4838	1.9372	0.3668
Total ${}^{2}$	105	992	7	9.51	89.86	0.63	0.54	0.46	p<0.001	0.4968	1.9873	0.3734

${}^{1}$ Fisher's exact test was used to evaluate the HWE because of the low number of individuals (Jerseys) per genotype (genotype counts below 5). p<0.01 ; p<0.001 – not consistent with the HWE. ${}^{2}$ Frequencies and parameters were calculated based on the total number of animals ( n=1104 ).

### 3.3 The association analysis

Table 3 shows the results of the association between the *DGAT1* K232A genotypes and milk production traits in the Jersey breed. The present analysis indicated that the cows carrying the KA genotype had a higher TDMY than those with the KK genotype. Moreover, cows carrying the KK genotype showed a higher FC and PC than those with the KA genotype in Jersey cows ( p<0.05 ). It is important to note that there were no Jersey cows with the AA genotype in this study (Table 3). Concerning the Holstein Friesian breed, the effect of the *DGAT1* genotypes was statistically significant only for the FC ( p<0.001 ). The KK genotype exhibited remarkably higher FC means compared to AA and heterozygotes. Although this genotype showed a lower TDMY and 305-DMY, the LSM analysis did not substantiate this association at the p<0.05 level (Table 4). Nevertheless, the effect of this genotype can be accepted as a tendency ( p<0.1 ). In herd 3, we evaluated the association between the reproduction traits (NI and CE), but the statistical analysis showed no significance in Holsteins (Table S1).

As shown in Table 5, the dominance effect ( d ) value of the *DGAT1* K232A for TDMY ( p<0.01 ), FC ( p<0.001 ), and PC ( p<0.005 ) was significant in Jersey cows. However, allele substitution effect ( a ) values were not significant ( p>0.05 ). Concerning the Holstein Friesian breed, a highly substantial additive effect ( a ) was observed for FC ( p<0.001 ). Dominance effect ( d ) on all milk production traits was insignificant ( p>0.05 ). On the other hand, a significant allele substitution effect ( α ) for FC ( p<0.001 ) and protein yield (PY; p<0.0 5) was detected in Holstein Friesian cows (Table 6).

**Table 3:** Levels of significance, least-squares means, and standard errors for the effect of *DGAT1* K232A genotypes on milk production traits in Jersey cows ( n=276 ).

Genotype	n	TDMY (kg d ${}^{-1}$ )	305-DMY (kg)	FC (%)	PC (%)	TDFY (kg d ${}^{-1}$ ) ${}^{1}$	TDPY (kg d ${}^{-1}$ ) ${}^{1}$	305-DFY (kg) ${}^{2}$	305-DPY (kg)
KA	227	16.07 ± 3.16 ${}^{a}$	5170.9 ± 1379.2	4.93 ± 0.58 ${}^{b}$	3.37 ± 0.17 ${}^{b}$	0.79 ± 0.15	0.54 ± 0.10	312.71 ± 11.00	275.47 ± 8.32
KK	49	14.96 ± 3.60 ${}^{b}$	4891.2 ± 1338.4	5.38 ± 0.71 ${}^{a}$	3.46 ± 0.18 ${}^{a}$	0.80 ± 0.20	0.52 ± 0.13	330.90 ± 19.32	259.42 ± 14.50

${}^{a,b}$ Different letters in a column indicate a significant difference at the p<0.05 . TDMY: test-day milk yield, 305-DMY: 305 d milk yield, FC: milk fat content, TDFY: test-day fat yield, PC: milk protein content, TDPY: test-day protein yield, 305-DFY: 305 d fat yield, 305-DPY: 305 d protein yield. ${}^{1}$ TDFY and TDPY (kg d ${}^{-1}$ ) were calculated based on TDMY. ${}^{2}$ p<0.1 ; a suggestive association (tendency).

## 4 Discussion

Fat and protein are essential milk components and are one of the main reasons for the nutritious property of milk. Milk fats are primarily made up of triglycerides, including one molecule of glycerin and three fatty acids. They are remarkable indicators of milk quality (Pathak et al., 2022). Depending on the fat level, milk has high concentrations of vitamins A and D (Magan et al., 2021). The nutritional value of milk and dairy products is closely associated with the fat content of milk, making up approximately 30 % of the total fat consumed in the human diet (Mansbridge and Blake, 1997; Pathak et al., 2022). Considering cattle milk is the most consumed milk type worldwide, molecular studies on cattle milk composition traits reveal crucial knowledge about human health and nutrition, primarily because of some fatty acids such as phospholipids (Pathak et al., 2022). Many researchers pointed out the *DGAT1* as a critical candidate and functional gene for fat traits and milk production (Gothwal et al., 2022; Grisart et al., 2002; Mahmoudi and Rashidi, 2023; Pathak et al., 2022). Several nucleotide alterations in this eminent gene have been associated with critical phenotypic traits in different breeds. Among them, K232A polymorphism (rs109234250) is one of dairy cattle's most famous genetic markers. However, the results of previously published reports are often conflicting. Moreover, there are some conspicuous limitations in these reports, such as limited sample sizes, lack of some genotypes (especially AA), and ignoring some crucial environmental factors in statistical models. In this report, we thoroughly analyze the effects of *DGAT1* K232A polymorphism on milk yield and quality in a sizeable population of commercial dairy cattle.

**Table 4:** Levels of significance, least-squares means, and standard errors for the effect of *DGAT1* K232A genotypes on milk production traits in Holstein Friesian cows ( n=828 ).

Genotype	n	TDMY (kg d ${}^{-1}$ )?	305-DMY (kg) ${}^{2}$	FC (%)	PC (%)	TDFY (kg d ${}^{-1}$ ) ${}^{1}$	TDPY (kg d ${}^{-1}$ ) ${}^{1}$	305-DFY (kg)	305-DPY (kg)
AA	7	25.48 ± 2.25	7812.41 ± 825.01	3.47 ± 0.26 ${}^{b}$	3.02 ± 0.11	0.97 ± 0.19	0.97 ± 0.19	284.55 ± 35.71	251.54 ± 26.60
KA	765	22.53 ± 0.96	7292.35 ± 343.21	3.84 ± 0.12 ${}^{b}$	3.16 ± 0.05	0.90 ± 0.22	0.90 ± 0.22	292.54 ± 15.81	245.80 ± 11.88
KK	56	21.12 ± 1.23	6814.18 ± 419.00	4.24 ± 0.14 ${}^{a}$	3.18 ± 0.06	0.90 ± 0.19	0.90 ± 0.19	301.00 ± 12.91	236.30 ± 14.34

${}^{a,b}$ Different superscripts within a column indicate significant differences at the p<0.001 level. TDMY: test-day milk yield, 305-DMY: 305 d milk yield, FC: milk fat content, TDFY: test-day fat yield, PC: milk protein content, TDPY: test-day protein yield, 305-DFY: 305 d fat yield, 305-DPY: 305 d protein yield. ${}^{1}$ TDFY and TDPY (kg d ${}^{-1}$ ) were calculated based on TDMY. ${}^{2}$ p<0.1 ; a suggestive association (tendency).

Concerning Jersey cows, the effects of *DGAT1* genotypes on TDMY ( p<0.01 ), FC ( p<0.001 ), and PC ( p<0.01 ) were statistically significant (Table 3). The cows with the KA genotype had more favorable values for TDMY ( + 1.11 kg d ${}^{-1}$ ) than cows with the KK genotype. We observed that the KK genotype of the *DGAT1* gene was related to higher FC ( + 0.45 %) and PC ( + 0.09 %) compared to the KA genotype. Although there were evident differences in the average 305-DMY, fat yield (FY), and PY, they were insignificant ( p>0.05 ). In this study, no Jersey cows with the AA genotype were found (Table 2). Anton et al. (2012) found that the AA-genotype animals had higher values of FC and PC than the other genotypes (5.83 % and 3.96 %, respectively) in Jersey cows. However, these animals exhibited low values for 305-DMY. Bovenhuis et al. (2016) reported that significant effects on milk protein composition in Dutch Holstein Friesians could not be confirmed in Danish Jersey or Danish Holstein Friesian breeds. Komisarek et al. (2004) found that the K allele was associated with high milk fat yield and fat and protein content, whereas the A allele led to increased milk yield in Jersey cows. It is important to note that we observed a lower frequency of the A allele in Jersey cattle. This finding is consistent with earlier reports (Kaupe et al., 2004; Winter et al., 2002; Krovvidi et al., 2021), which indicate that a high frequency of the *DGAT1* K allele is a characteristic feature of Jersey cattle. Notably, Ripoli et al. (2006) demonstrated that breeds involved in different lineages affect the genotypic distributions. They stated that European *Bos taurus* breeds, except the Jersey breed, showed the lowest frequency of the K allele, while cattle of the *Bos indicus* type harbored the highest K allele frequencies. This situation is one of the most significant reasons for the contradictory results from different breeds regarding milk production traits. It is well known that gene frequencies may differ among different breeds or even different populations of the same breed (Ardicli et al., 2019a). Hence, genotypic results from sizeable dairy populations may reveal more reliable results on genotype–phenotype associations. Several studies have reported that the *DGAT1* K232A marker influences milk yield and composition at the same time. Here, we note that this polymorphism impacted the FC and PC but not 305-DMY. Furthermore, the statistically significant effect of the *DGAT1* marker turned out into a tendency ( p<0.1 ) only for the fat yield when we expanded the analysis to 305 d lactation (for six lactations). This result again shows how comprehensive data are a vital prerequisite for reliably evaluating the association between single-nucleotide polymorphisms (SNPs) and the milk production traits in commercial dairy herds.

**Table 5:** Genetic effects of *DGAT1* K232A polymorphism on milk yield and milk component traits in Jersey breed.

Traits	Additive	Dominant	Allele substitution
	effect ( a )	effect ( d )	effect ( α )
TDMY (kg)	7.48	8.59 ${}^{**}$	6.9646
305-DMY (kg)	2445.6	2662.3	2285.862
Fat %	2.69	2.24 ${}^{***}$	2.5556
Fat yield (kg d ${}^{-1}$ )	0.4	0.39	0.3766
Protein %	1.73	1.64 ${}^{**}$	1.6316
Protein yield (kg d ${}^{-1}$ )	0.26	0.28	0.2432

TDMY: test-day milk yield; 305-DMY: 305 d milk yield. Fat and protein yields (kg) were calculated based on TDMY. ${}^{**}$ p<0.01 . ${}^{***}$ p<0.001 .

*DGAT1* is an efficient and functional gene in which alterations may affect several essential traits in dairy cattle. Many authors stated that *DGAT1* K232A polymorphism influenced milk yield (Anton et al., 2008; Bovenhuis et al., 2016; Li et al., 2021; Manga et al., 2011; Mao et al., 2012; Molee et al., 2015; Schennink et al., 2007; Sun et al., 2009; Vanbergue et al., 2016). We found no significant association between the *DGAT1* marker and milk yield in either of the dairy breeds. Similar results for FC and differences for 305-DMY and PC in Holstein cows were also observed by Anton et al. (2012). Lešková et al. (2013) reported that genotype significantly affected PC but not for 305-DMY and FC in Holstein cows. Contrary to some papers in the literature, we found that the AA-genotype cows tended ( p<0.1 ) to exhibit higher 305-DMY than those with alternative genotypes (Table 4). However, this difference was not corroborated by the statistical analysis. It is important to note that we observed only seven animals carrying this genotype. In general, the frequency of this genotype is remarkably low or absent in Holstein Friesians (Ardicli et al., 2018, 2019b). Hence, we think the association results presented in this study may be helpful for further investigations.

**Table 6:** Genetic effects of *DGAT1* K232A polymorphism on milk yield and milk component traits in Holstein Friesian breed.

Traits	Additive	Dominant	Allele substitution
	effect ( a )	effect ( d )	effect ( α )
TDMY (kg)	- 2.18	- 0.77	5.5372
305-DMY (kg)	- 499	- 20.65	- 499.23
Fat %	2.505 ${}^{***}$	- 0.015	0.4511 ${}^{***}$
Fat yield (kg d ${}^{-1}$ )	- 0.035	- 0.035	- 0.0329
Protein %	0.08	0.006	0.08096
Protein yield (kg d ${}^{-1}$ )	- 0.1 ${}^{2}$	- 0.05	- 0.097 ${}^{*}$

TDMY: test-day milk yield; 305-DMY: 305 d milk yield. Fat and protein yields (kg) were calculated based on TDMY. ${}^{*}$ p<0.05 . ${}^{**}$ p<0.01 . ${}^{***}$ p<0.001 .

The effect of genotype on FC in the present study was highly significant ( p<0.001 ). The KK genotype had a higher FC ( + 0.77 % and + 0.40 %) than AA and KA. The K allele was associated with higher FC. A recent meta analysis by Mahmoudi and Rashidi (2023) reached similar results for the impact of the K232A marker on FC. These researchers concluded that the K allele tremendously increased milk FC, especially when two copies of this allele are inherited together. In contrast, the A allele of K232A polymorphism had adverse effects. This suggestion which ensued from an extensive statistical aspect by Mahmoudi and Rashidi (2023) has been experimentally substantiated by the present study. However, they found that the K232A marker influenced the PC as well. On the contrary, we observed no statistical difference among the genotypes for the PC in the Holstein Friesian breed (Table 4).

In short, Holstein cows with the AA genotype had higher TDMY ( p<0.1 ) and 305-DMY ( p<0.1 ) but significantly lower FC ( p<0.001 ). Similar results for 305-DMY yields in Holstein cows have also been reported by Sun et al. (2009). Conversely, the same research noted that FC and PC were not different according to genotypes for the *DGAT1* marker. The current results agreed with that of Kadlecová et al. (2014) in which AA-genotype animals had the highest milk yield and the lowest FC. Several researchers also pointed out the association between *DGAT1* K232A and FC (Carvajal et al., 2016; Li et al., 2021; Manga et al., 2011; Mao et al., 2012; Molee et al., 2015; Schennink et al., 2007; Vanbergue et al., 2016). Unlike the current study, Anton et al. (2012) observed that AA-genotype cows had lower 305-DMY (8247 kg) but high FC (4.34 %) and PC (3.41 %) in Holstein cows. The finding disagrees with the report of Dokso et al. (2015), who observed that the KK genotype (9224.6 kg) was found to be higher than the KA (8866.2 kg) and AA (8825.8 kg) genotypes. However, this difference was not statistically significant. Sun et al. (2009) reported that FC and PC have not differed between genotypes determined for the *DGAT1* gene. Tumino et al. (2021) also indicated that K232A polymorphism did not influence the FC. The *DGAT1* K232A marker has been shown to be a strong candidate for determining milk production and components. However, the details about the association analysis results and the types of influenced traits may vary widely.

In addition to milk production characteristics in dairy cattle, another critical issue is reproductive performance. Purely yield-oriented selection over many years has resulted in a significant decrease in fertility in dairy cattle. Selection programs supported by current studies include important reproductive characteristics in the indexes (Ardicli et al., 2024; Ooi et al., 2024). Hence, the impact of the *DGAT1* gene on bovine reproduction is a crucial assessment in dairy cattle. Rychtarova et al. (2014) and Ardicli et al. (2019b) reported that *DGAT1* had a significant effect on calving interval. Demeter et al. (2009) found a potential impact of the *DGAT1* on non-return rates for insemination 28 and 56 d after the first service. Collis et al. (2012) found a significant association between the *DGAT1* K232A polymorphism and age at puberty. In this study, we evaluated NI and CE in Holstein Friesian cows but did not observe any significant phenotype–genotype relation (Table S1). Berry et al. (2010) also indicated no association of the K232A marker with fertility traits and calving performance in Irish Holstein Friesians. It is important to note that the current knowledge on the influence of *DGAT1 *K232A marker on reproduction performance is relatively low, especially compared to milk production traits. Hence, it strongly needs further research.

We have observed a salient effect ( p<0.001 ) of *DGAT1* K232A polymorphism on the FC in both dairy breeds. Concerning the present analysis, the AA genotype constituted only ∼ 1 % of the total genotype. In many genetic studies, excluding the rare variant is a common implementation in statistical analyses. Hence, we excluded the AA genotype and tested the observed associations subsequently in Holstein Friesians (data not shown). This implementation did not impair the significance of the FC. The dominance effect of *DGAT1* K232A for TDMY, FC, and PC was significant in Jersey cows (Table 5). However, there were no significant results for allele substitution effect. A very similar result was presented by Anton et al. (2012), indicating a significant dominance effect on FC and PC in Jersey cows raised in Hungary ( p<0.05 ).

Very recent studies particularly emphasize the importance of this gene in dairy cattle. Overexpression of *DGAT1* in adipocytes treated with epinephrine decreased both lipolysis and autophagy, while silencing *DGAT1* intensified these processes. Collectively, these results suggest that increasing bovine *DGAT1* levels might serve as a regulatory mechanism to inhibit fat breakdown in adipocytes, emphasizing its role in sustaining metabolic balance in dairy cows during energy deficits (Xu et al., 2024). Interestingly, this gene was identified as a positional candidate gene for milk urea (Atashi et al., 2024). The genomic area containing *DGAT1* K232A polymorphism is highly dynamic and crucial for several key metabolic processes, including fat and energy balance. In a recent study by Tang et al. (2024), linkage disequilibrium analysis revealed that SNP rs109421300 is closely linked with the K232A mutation in the *DGAT1* gene, and it remains an independent gene expression quantitative trait loci (eQTL) even when controlled for in-gene expression studies. Thus, rs109421300 could be a critical marker influencing *DGAT1* gene expression, alternative splicing, and various phenotypic traits, warranting further investigation. Archetypal clustering analysis identified a cluster with an archetypal variant near the *DGAT1* gene, including variants linked to *CDH2*, *BTRC*, *SFRP2*, *ZFHX3*, and *SLITRK5*. This cluster seems to influence milk yield but has minimal impact on fertility. These genes are associated with insulin, adipose tissue, and energy metabolism (Ooi et al., 2024). These studies unequivocally underscore the necessity for further elucidation of critical biological aspects associated with the *DGAT1* gene, highlighting the significance of this genomic region in the genetics of dairy cattle.

Regarding the characterization of the genetic effects of the *DGAT1* K232A marker on milk yield and milk component traits in the Holstein Friesian breed, the additive effect for FC was highly significant, while no significant dominance effect was observed. The allele substitution effect for FC was also significant (Table 6). This finding was consistent with the results of Koopaei et al. (2012), who observed that the additive effect on FC was significant ( + 0.455 %; p<0.001 ). Similarly, Sun et al. (2009) reported the additive effect and allele substitution effect on PY to be 4.67 ( p<0.01 ) and 5.14 ( p<0.01 ). However, they reported no significant additive, dominance, or allele substitution effects for FC and PC. Anton et al. (2012) observed the additive effects on 305-DMY (642.8 kg; p<0.05 ) and PC (0,091 %; p<0.05 ). They also reported a significant additive effect ( p<0.05 ) on FC (0.31 %) and a dominance over 305-DMY (189.2 kg), FC ( - 0.253 %), and PC ( - 0.045 %). Krovvidi et al. (2021) demonstrated that the least-squares mean of milk yields over lactations was significantly higher ( p<0.05 ) in homozygous (AA) genotypes of both Jersey and Holstein Friesian crossbred cattle after adjusting for farm, parity, and season effects. However, the fat, solids-not-fat (SNF), and protein content in AA genotypes was lower than those in KK genotypes across both genetic groups, though not significantly ( p>0.05 ). Samuel et al. (2023) reported that the AA genotype was linked to higher milk yields in Boran × Holstein Friesian crosses, while the KA genotype was associated with increased yields in zebu population ( p<0.05 ). Milk from KA genotype cattle exhibited lower fat and lactose contents compared to the KK genotype across all genetic groups ( p<0.05 ). Furthermore, replacing one K allele with an A allele significantly reduced daily milk yield by up to 3 L and lactose content by 0.58 %, but increased fat content by up to 0.81 % in the populations studied, underscoring the impactful role of the DGAT1 *K232A* marker on milk production traits (Samuel et al., 2023).

Our results indicated that the most striking feature of the *DGAT1* K232A polymorphism is the highly significant additive effect on the FC. Although previously published reports have pointed out a potential effect of this marker on yield traits, here we report that *DGAT1* K232A should be focused on considering milk quality parameters primarily for the fat content of cattle milk. However, it needs further investigation, mainly supported by genotypic interactions and epigenetic dynamics.

## 5 Conclusions

This paper extensively focuses on the effects of the *DGAT1* K232A polymorphism concerning milk production traits in dairy cattle. Significant differences exist between the genotypes of KA and KK belonging to the *DGAT1* marker on test-day milk yield, fat, and protein contents in the Jersey cows. In this breed, the heterozygous genotype had higher test-day milk yield but lower fat and protein contents. Regarding the Holstein Friesian breed, we found significant differences among genotypes on milk fat content. Remarkably, Holstein Friesians cows that possess the KK genotype displayed significantly higher milk fat content means compared to both AA-genotype cows and heterozygotes. Similarly, Jersey cows with the KK genotype demonstrated enhanced milk fat content and, moreover, milk protein content relative to those with the heterozygous genotype. Consequently, we comprehensively investigate the effects of the *DGAT1* K232A marker on milk yield and quality in dairy herds of Holstein Friesian and Jersey cows. Consideration of this marker on the development process of the milk quality of cows will provide much more reliable results in a shorter period. This study confirmed the results of some studies in the literature, and particular contradictory results were observed. Furthermore, this study has yielded valuable data for genetic evaluations aimed at enhancing milk content and offers reliable, detailed, and comparative insights into *DGAT1* K232A, a critical genetic marker in dairy cattle, across two prevalent dairy breeds.

## Supplement

10.5194/aab-67-455-2024-supplement The supplement related to this article is available online at: https://doi.org/10.5194/aab-67-455-2024-supplement.

## Data Availability

The datasets generated are available from the corresponding author on request.
