# Supplementary material for: A comprehensive analysis of the effects of DGAT1 K232A polymorphism on milk production and fertility traits in Holstein Friesian and Jersey cows reared in Türkiye
Source: Arch Anim Breed. 2024 Oct 2;67(4):455–67. doi: 10.5194/aab-67-455-2024 (PMC13196880; doi:10.5194/aab-67-455-2024)
Supplement: The supplement related to this article is available online at: https://doi.org/10.5194/aab-67-455-2024-supplement. [file aab-67-455-2024-supplement.pdf]

Supplement of Arch. Anim. Breed., 67, 455–467, 2024
https://doi.org/10.5194/aab-67-455-2024-supplement
© Author(s) 2024. CC BY 4.0 License.

Open Access
Archives Animal Breeding

Supplement of

A comprehensive analysis of the effects of *DGAT1* K232A polymorphism on milk production and fertility traits in Holstein Friesian and Jersey cows reared in Türkiye

Sena Ardicli et al.

Correspondence to: Ozden Cobanoglu (ocobanoglu@uludag.edu.tr)

The copyright of individual parts of the supplement might differ from the article licence.

Table S1. Levels of significance, least-squares means, and standard errors for the effect of *DGAT1* K232A genotypes on fertility traits in Holstein-Friesian cows (*n*=361).

**Traits**	**Genotype**		
	**KA**	**KK**	***p*-value**
**Number of inseminations**[1]	2.27±0.07	2.20±0.29	0.820
**Calving ease**[2]	2.20±0.06	2.18±0.32	0.955

[1]Number of inseminations per conception

[2]Calving ease was assessed using a scale based on the following criteria: 1 = unassisted calvings; 2 = calvings requiring minor assistance; 3 = calvings requiring hands-on help; and 4 = calvings that necessitated Caesarean sections. Due to a low number of animals in category 4, categories 3 and 4 were merged and recoded as 3 to address the issue of sparse data in the most severe category.
